# The S273R protein of African swine fever virus antagonizes the canonical NF-*κ*B signaling pathway by I*κ*B*α*

**DOI:** 10.1128/jvi.02225-24

**Published:** 2025-03-31

**Authors:** Haojie Ren, Lan-Fang Shi, Yanjin Wang, Xiao-Ya Pan, Su Li, Yu-He Ma, Jun-Hao Fan, Xing Chen, Zhong-Yuan Yang, Shuai Fan, Yuhang Zhang, Shichong Han, Wen-Rui He, Bo Wan, Hua-Ji Qiu, Gai-Ping Zhang

**Affiliations:** 1 International Joint Research Centre of National Animal Immunology, College of Veterinary Medicine, Henan Agricultural University 731518, Zhengzhou, Henan, China; 2 State Key Laboratory for Animal Disease Control and Prevention, National African Swine Fever Para-Reference Laboratory, National High-Containment Facilities for Animal Disease Control and Prevention, Harbin Veterinary Research Institute, Chinese Academy of Agricultural Sciences 12661, Harbin, Heilongjiang, China; 3 Longhu Laboratory 693032 , Zhengzhou, Henan, China; 4 Ministry of Education Key Laboratory for Animal Pathogens and Biosafety, Henan Agricultural University 70573, Zhengzhou, Henan, China; Lerner Research Institute, Cleveland Clinic, Cleveland, Ohio, USA

**Keywords:** African swine fever virus, canonical NF-*κ*B signaling pathway, S273R protein, I*κ*B*α*, inflammatory responses

## Abstract

African swine fever virus (ASFV) is a large double-stranded DNA virus, which is the causative agent of African swine fever (ASF), a devastating disease of suids epidemic in many countries. The virus has developed multiple strategies to evade surveillance from the host immune system. Inflammatory responses, especially the NF-*κ*B signaling pathway, play central roles in ASFV pathogenesis and immunoevasion. In this study, we identified the ASFV S273R protein (pS273R) as an antagonist of the canonical NF-*κ*B signaling pathway independently of its protease activity. The ectopically expressed pS273R markedly inhibited the tumor necrosis factor-alpha or interleukin-1 beta-triggered NF-*κ*B signaling pathway in HEK293T and PK-15 cells. Silencing pS273R by RNA interference led to elevated expression levels of proinflammatory cytokines in the ASFV-infected primary porcine alveolar macrophages. Mechanistically, pS273R functioned independently of its protease activity. pS273R was associated with the NF-*κ*B complex and interrupted the translocation of I*κ*B*α* into the proteasome, resulting in the increased stability of I*κ*B*α* and subsequently impaired nuclear translocation of p65. Furthermore, the core domain (amino acids 83–273) of pS273R was essential for the pS273R-mediated inhibition of the NF-*κ*B signaling pathway. These findings demonstrate the immunosuppressive role of pS273R and provide novel insights into ASFV biological characteristics.

**IMPORTANCE:**

African swine fever (ASF) is a hemorrhagic disease of suids caused by African swine fever virus (ASFV), with morbidity and mortality rates of up to 100%. The disease has led to significant economic losses to the global swine industry. In this study, we identify the ASFV S273R protein (pS273R) as an antagonist of the canonical NF-*κ*B signaling pathway. Our findings demonstrate the immunosuppressive role of pS273R, which will contribute to a better understanding of the pathogenesis of ASFV and may contribute to the development of antiviral therapies against ASF.

## INTRODUCTION

African swine fever (ASF) is a hemorrhagic disease of suids caused by African swine fever virus (ASFV), with a mortality of up to 100% for virulent strain infection. The disease has led to significant economic losses to the global swine industry (1–4). As the sole member of the *Asfarviridae* family, ASFV has a complex multi-layered virion structure with a double-stranded DNA genome, which is approximately 170–194 kilo-base pairs (kb) in length and encodes more than 160 viral proteins. However, the structure and functions of many viral proteins are yet to be clarified to date, which restricts the development of ASF vaccines. In the past decade, several promising vaccine candidates, such as naturally attenuated Lv17/WB/Rie1, have been developed and evaluated. Unfortunately, these viruses were not able to induce protective immunity in pigs against a virulent strain challenge (5). No commercial ASF vaccines are available worldwide except in Vietnam, where the Vietnam ASF vaccine ASFV-G-ΔI177L efficiently protects European and native pig breeds against circulating Vietnamese field strains (6, 7). Further in-depth studies of the mechanisms underlying ASFV pathogenesis would facilitate the development of ASF vaccines.

ASFV infection increases the secretion of proinflammatory cytokines, such as tumor necrosis factor-alpha (TNF-*α*), interleukin-1 beta (IL-1*β*), IL-6, and IL-8 (8–11). The canonical nuclear factor kappa B (NF-*κ*B) signaling pathway plays key roles in both host antiviral immunity and ASFV pathogenesis (12). Once ASFV is recognized by pattern recognition receptors (PRRs), the secreted proinflammatory cytokines, including TNF-*α* or IL-1*β*, are sensed by the TNF receptor 1 (TNFR1) or IL-1 receptor (IL-1R) in the plasma membrane, followed by the recruitment and activation of adaptors, including the TNFR-associated death domain protein (TRADD), myeloid differentiation primary response 88 (MyD88), transforming growth factor beta-activated kinase 1 (TAK1), and TAK1-binding protein 1 (TAB1), ultimately activating the I*κ*B kinase beta (IKK*β*) (13). Next, the NF-*κ*B inhibitor alpha (I*κ*B*α*) is phosphorylated and ubiquitinated, leading to proteasomal degradation (14). Finally, the p65-p50 heterodimer is released from I*κ*B*α* and translocates to the nucleus, resulting in the transcription of various cytokines and subsequent inflammatory responses (15).

The activation of the NF-*κ*B signaling pathway can regulate various physiological processes, such as innate and adaptive immune responses, inflammation, and pyroptosis in host cells (16–18), which makes it an attractive target for viral immune invasion. Some proteins of ASFV, such as pMGF505-7R, pMGF300-4L, pH240R, pI10L, and pA238L, are involved in regulating the NF-*κ*B signaling pathway to evade the cellular antiviral immunity. pMGF505-7R inhibits the NF-*κ*B activation by binding to IKK*α*, leading to the reduced production of IL-1*β* (8). pMGF300-4L interacts with both IKK*β* and I*κ*B*α*, resulting in the inhibition of nuclear translocation of p65 and the production of IL-1*β* and TNF-*α* (19). Due to the aberrant virion morphogenesis and enhanced inflammatory cytokine expression, deletion of the *H240R* gene decreases the production of infectious viral progenies (20). pI10L also participates in regulating the TNF-*α*- or IL-1*β*-triggered NF-*κ*B signaling pathway by targeting IKK*β*, followed by the reduced expression levels of inflammatory cytokines (21). pA238L is an anti-inflammatory protein, and its structure is similar to I*κ*B*α*, which can inhibit the NF-*κ*B activation and therefore suppress the host inflammatory responses (22). Among them, recent studies have highlighted the roles of pMGF300-4L, pMGF505-7R, and pH240R in viral pathogenicity, underscoring the significance of inflammatory responses during ASFV infection (19, 23). Further in-depth studies on the mechanisms underlying the regulation of inflammatory responses of ASFV proteins could facilitate the understanding of this deadly virus.

The structural protein pS273R, encoded by the *S273R* gene, is located in the core-shell of ASFV virions (24). As a cysteine protease, pS273R is a member of the small ubiquitin-like modifier 1 (SUMO1)-specific protease family (25) and has two domains, that is, the core domain and the arm domain (26). Considering the indispensability of pS273R in the maturation of pp220 and pp62, pS273R becomes a desirable target for the therapeutic prevention of ASFV (27). pS273R is not only required for the maturation of viral structural proteins but also involved in viral evasion of host innate immune responses. It has been shown that pS273R can reduce the production of type I interferons (IFNs) by antagonizing the cGAS-STING signaling pathway by targeting IKK*ε* (28). Meanwhile, pS273R bridges the interaction between signal transducers and activators of transcription 2 (STAT2) and the E3 ubiquitin ligase DCST1, which induces the degradation of STAT2 and impairs the type I IFNs-mediated antiviral responses (29). In addition, pS273R could cleave the GTPase-activating protein (SH3 domain)-binding protein 1 (G3BP1), vital stress granules (SGs)-nucleating protein, which impaired SGs formation and relieved the inhibitory effect of SGs on ASFV replication (30). pS273R also inhibits pyroptosis by noncanonical cleavage of gasdermin D (GSDMD) to promote ASFV replication (31). Therefore, pS273R plays multiple roles in the ASFV life cycle. However, its involvement in the regulation of the ASFV-triggered inflammatory responses remains elusive.

In a previous study, we screened a total of 179 proteins of ASFV that were able to regulate the canonical NF-*κ*B signaling pathway, among which, pS273R could inhibit the TNF-*α*-triggered activation of the NF-*κ*B promoter (21). In this study, we demonstrated that pS273R remarkably inhibited the TNF-*α*- or IL-1*β*-induced transcription levels of proinflammatory cytokines. Knockdown of *S273R* induced the elevated production level of IL-1*β* in the ASFV-infected porcine primary alveolar macrophages (PAMs). Mechanistic study suggested that pS273R bound to the NF-*κ*B complex (I*κ*B*α*, p65, and p50) and enhanced the stability of I*κ*B*α* by hindering the association of I*κ*B*α* with the proteasome, thus impairing the expression levels of proinflammatory cytokines. This study elucidates the function of pS273R in regulating the inflammatory response after ASFV infection and provides a novel theoretical basis for understanding the biological characteristics of ASFV. These efforts will contribute to the development of vaccines and antivirals against ASF.

## RESULTS

### The ASFV pS273R inhibits the TNF-α- or IL-1β-triggered activation of the canonical NF-κB signaling pathway

**Fig 1:**
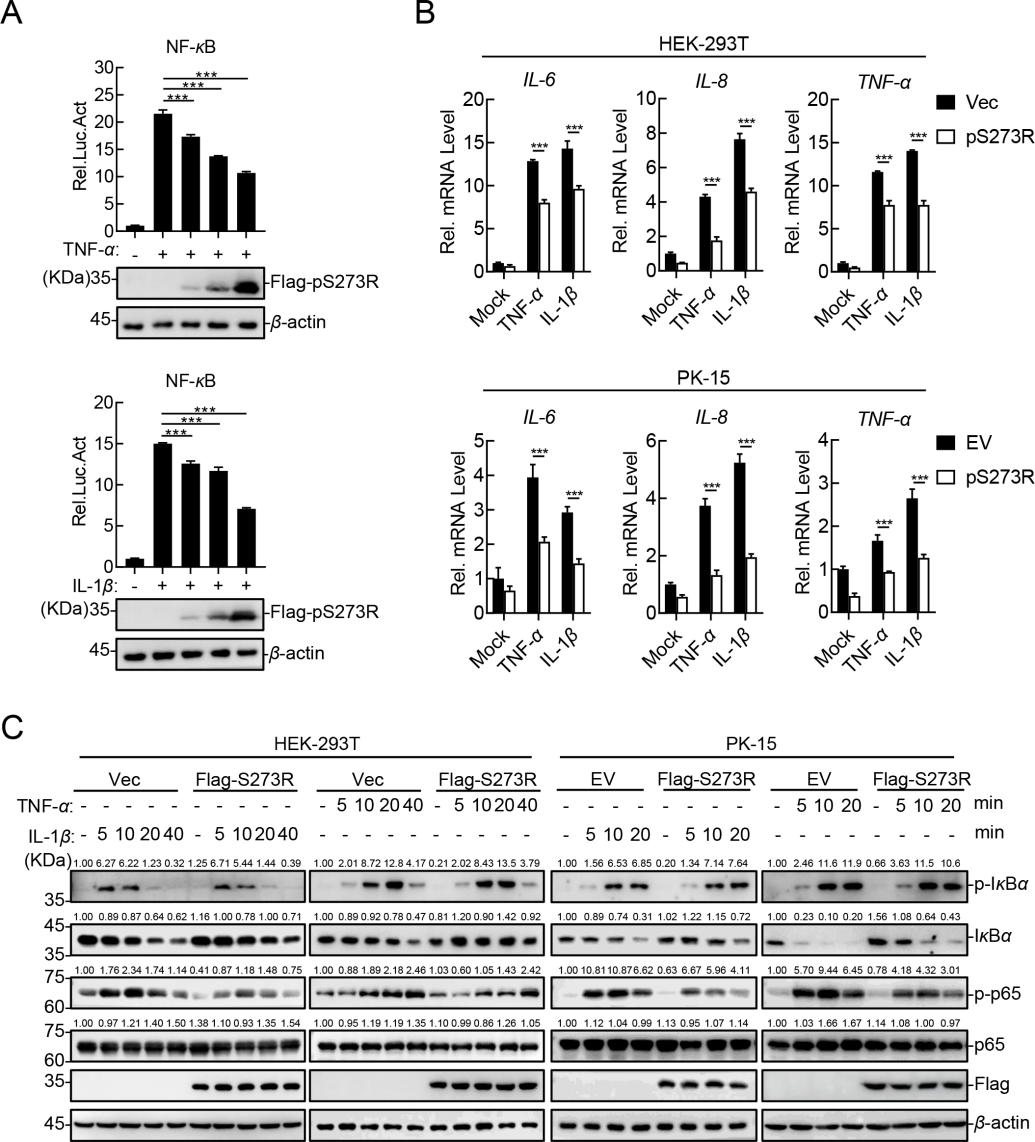
The ASFV pS273R inhibits the TNF-*α*- or IL-1*β*-triggered NF-*κ*B signaling pathway. (**A**) The ASFV pS273R inhibits the TNF-*α*- or IL-1*β*-triggered activation of the NF-*κ*B promoter in a dose-dependent manner. HEK293T cells were cotransfected with the different concentrations of the pS273R-expressing plasmid, pNF-*κ*B-Fluc, and pRL-TK. At 24 h post-transfection (hpt), the cells were treated with 20 ng/mL TNF-*α* or IL-1*β*, followed by luciferase assay and western blotting analysis. (**B**) The ASFV pS273R inhibits the TNF-*α*- or IL-1*β*-triggered transcription levels of proinflammatory cytokines. The HEK293T cells transfected with pFlag-S273R or the PK-15 cells stably expressing pS273R were treated with TNF-*α* or IL-1*β*, and the total RNA was extracted for RT-qPCR assay. (**C**) The ASFV pS273R inhibits the TNF-*α*- or IL-1*β*-triggered p65 phosphorylation. HEK293T cells transfected with pFlag-S273R or the PK-15 cells stably expressing pS273R were treated with TNF-*α* or IL-1*β* for the indicated hpt. Western blotting analysis was performed using the indicated antibodies. The densitometric analysis of the protein expression levels was performed using ImageJ software. Ratio: target protein/*β*-actin. The data shown were the means ± SDs from one representative experiment performed in triplicates (**A and B**). *P < 0.05; **P < 0.01; ***P < 0.001; and ns, not significant (*P* > 0.05) (unpaired Student’s *t* test).

We have previously shown that pS273R could inhibit the TNF-*α*-induced activation of the NF-*κ*B promoter (21), which indicates that pS273R may be an antagonist in the ASFV-triggered inflammatory responses. To confirm the role of pS273R in the activation of the canonical NF-*κ*B signaling pathway, HEK293T cells were transfected with different concentrations of the pS273R-expressing plasmid and lysed, followed by a dual-luciferase reporter assay and western blotting analysis. The results showed that pS273R inhibited the TNF-*α*- or IL-1*β*-triggered activation of the NF-*κ*B promoter in a dose-dependent manner (Fig. 1A). Next, the transcription levels of several proinflammatory cytokines in the pS273R-overexpressing HEK293T or PK-15 cells treated with TNF-*α* or IL-1*β* were measured. The RT-qPCR results showed that the ectopically expressed pS273R remarkably inhibited the mRNA transcription levels of the *IL-6*, *IL-8*, and *TNF-α* genes induced by TNF-*α* or IL-1*β* (Fig. 1B). Furthermore, western blotting analysis showed that the phosphorylation of I*κ*B*α* and p65 induced by TNF-*α* or IL-1*β*, which were the hallmarks in the activation of the canonical NF-*κ*B signaling pathway, were remarkably inhibited in the pS273R-overexpressing HEK293T cells compared with the pRK-transfected cells (Fig. 1C). Consistently, pS273R also markedly reduced the phosphorylation of I*κ*B*α* and p65 in PK-15 cells (Fig. 1C). These results demonstrate that pS273R negatively regulates the production of the TNF-*α*- or IL-1*β*-induced proinflammatory cytokines in both HEK293T and PK-15 cells, and pS273R can inhibit the canonical NF-*κ*B signaling pathway triggered by TNF-*α* or IL-1*β*.

### Knockdown of the ASFV pS273R results in enhanced activation of the canonical NF-κB signaling pathway

**Fig 2:**
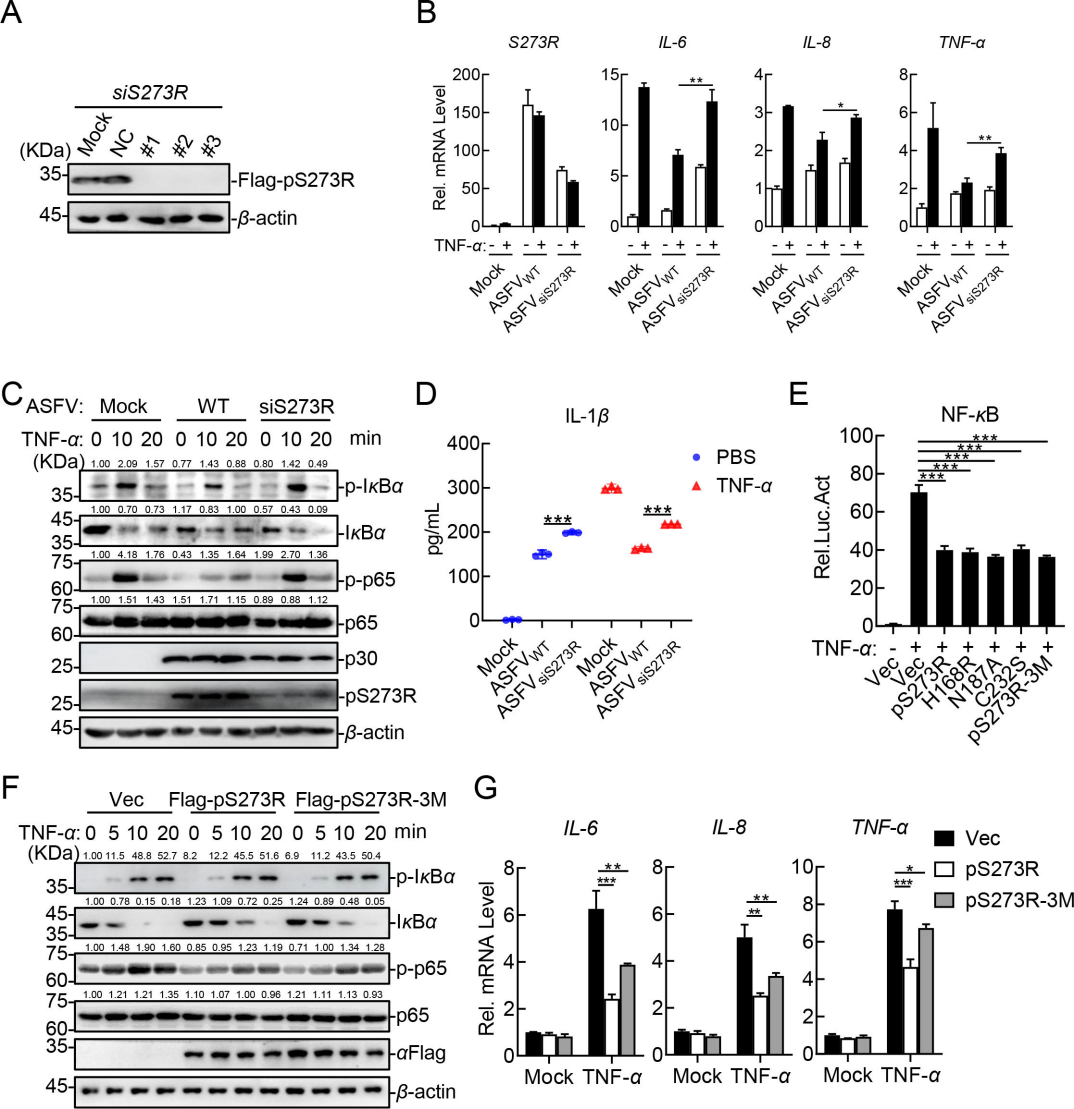
The ASFV pS273R inhibits the NF-*κ*B signaling pathway independently of its protease activity. (**A**) Knockdown efficiency of the *S273R*-specific siRNAs. The *S273R*-specific siRNAs were transfected into the HEK293T cells overexpressing pS273R, followed by western blotting analysis using the indicated antibodies. (**B**) Knockdown of *S273R* induces elevated transcription levels of proinflammatory cytokines in the ASFV-infected PAMs. PAMs were transfected with the siRNAs, followed by ASFV infection. At 24 h postinfection (hpi), the cells were treated with 20 ng/mL TNF-*α*, and then, total RNA was extracted for RT-qPCR assay. (**C**) Knockdown of *S273R* elevates the p65 phosphorylation in the ASFV-infected PAMs. PAMs were transfected with the siRNAs for 12 h and infected with ASFV for 24 h. After treatment with TNF-*α* or left untreated at the indicated hpi, western blotting analysis was performed using the indicated antibodies. (**D**) Knockdown of *S273R* increases the TNF-*α*-induced cytokine production in the ASFV-infected PAMs. PAMs were transfected with the siRNAs infected with ASFV and then treated with TNF-*α* or left untreated. The IL-1*β* expression level in the cell culture supernatants was detected using a commercial IL-1*β* ELISA kit. (**E**) pS273R mutants inhibit the TNF-*α*-triggered activation of the NF-*κ*B promoter. HEK293T cells were cotransfected with the NF-*κ*B reporter, pRL-TK, and pFlag-S273R or pFlag-S273R mutants. At 24 h post-transfection (hpt), the cells were treated with 20 ng/mL TNF-*α*, followed by luciferase assay and western blotting analysis. (**F**) pS273R-3M inhibits the TNF-*α*-triggered phosphorylation of p65. PK-15 cells stably expressing pS273R or pS273R-3M were treated with TNF-*α* for the indicated minutes. Subsequently, the cells were lysed, followed by western blotting analysis using the indicated antibodies. (**G**) pS273R-3M inhibits the TNF-*α*-triggered transcription levels of proinflammatory cytokines in PK-15 cells. PK-15 cells stably expressing pS273R or pS273R-3M were treated with TNF-*α*, and the total RNA was extracted for RT-qPCR assay. The densitometric analysis of the protein expression levels was performed using the ImageJ software. Ratio: target protein/*β*-actin (**C and F**). The data shown were the means ± SDs from one representative experiment performed in triplicates (B, D, E, and G). *P < 0.05; **P < 0.01; ***P < 0.001; and ns, not significant (*P* > 0.05) (unpaired Student’s *t* test).

To further characterize the functional role of pS273R in the regulation of the canonical NF-*κ*B signaling pathway, RNA interference (RNAi) was used to silence the expression of the *S273R* gene. The HEK293T cells overexpressing pS273R were transfected with the *S273R*-targeting siRNAs, and western blotting analysis showed that the siRNA could silence the expression of the *S273R* gene efficiently (Fig. 2A). Then, the transcription levels of several proinflammatory cytokines were measured by RT-qPCR. The results revealed that ASFV infection induced the production of *IL-6*, *IL-8*, and *TNF-α*, whereas upon treatment with TNF-*α*, ASFV infection significantly attenuated the production of these inflammatory cytokines compared with the control group. The siRNAs dramatically reduced the mRNA level of the *S273R* gene, and the knockdown of *S273R* led to elevated transcription levels of the *IL-6*, *IL-8*, and *TNF-α* genes in the ASFV-infected PAMs (Fig. 2B). Furthermore, western blotting analysis showed that the expression of S273R was markedly decreased due to siRNA treatment, and the phosphorylation of p65 was significantly reduced in the ASFV-infected PAMs consistently (Fig. 2C). To verify the production of cytokines at the protein level, the IL-1*β* production in the cell supernatants was measured by ELISA. The results showed that knockdown of *S273R* led to the elevated production of IL-1*β* in the PAMs infected with ASFV (Fig. 2D). These results indicate that pS273R plays an important role in the immunoevasion of ASFV.

### The inhibition of ASFV pS273R on the canonical NF-κB signaling pathway is independent of its protease activity

It has been shown that the catalytic triad H168-N187-C232 of pS273R is essential for its protease activity, and the mutation of H168, N187, or C232 results in the protease inactivity of pS273R (26). To explore whether the protease activity was essential for the inhibition of the canonical NF-*κ*B signaling pathway by pS273R, the protease-inactivated mutants (H168R/N187A/C232S) of pS273R designated as pS273R-H168R, pS273R-N187A, pS273R-C232S, and pS273R-3M were generated (26). Interestingly, consistent with pS273R, either pS273R-H168R, pS273R-N187A, pS273R-C232S, or pS273R-3M inhibited the TNF-*α*-triggered activation of the NF-*κ*B promoter (Fig. 2E). Western blotting analysis demonstrated that the phosphorylation of p65 was reduced in the cells stably expressing pS273R or pS273R-3M (Fig. 2F). Moreover, the RT-qPCR results showed that pS273R-3M exhibited similar inhibition of the TNF-*α*-induced transcription levels of the *IL-6*, *IL-8*, and *TNF-α* genes (Fig. 2G). These results suggest that pS273R inhibits the canonical NF-*κ*B signaling pathway independently of the protease activity of pS273R.

### The ASFV pS273R is associated with the NF-κB complex

**Fig 3:**
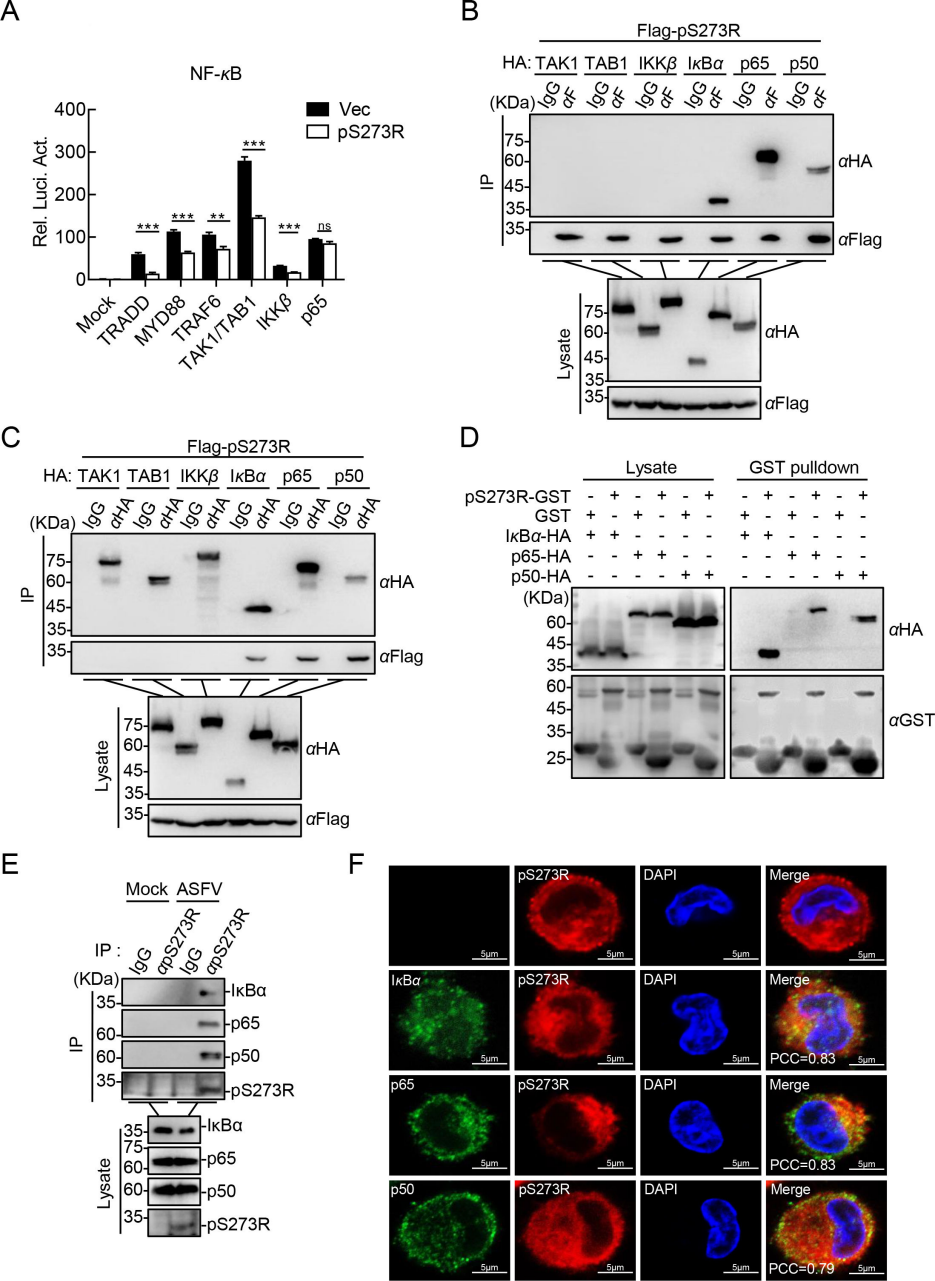
The ASFV pS273R is associated with the NF-*κ*B complex. (**A**) ASFV pS273R functioned at or upstream of p65. HEK293T cells were cotransfected with pNF-*κ*B-Fluc, pRL-TK, and the indicated expression plasmids, followed by luciferase assay. (**B and C**) ASFV pS273R interacts with I*κ*B*α*, p65, or p50. HEK293T cells were cotransfected with the plasmids expressing the HA-TAK1, -TAB1, -IKK*β*, -I*κ*B*α*, -p65, or -p50, and pFlag-S273R, and then lysed for co-IP using anti-HA or anti-Flag MAb, followed by western blotting analysis using the indicated antibodies. (**D**) The ASFV pS273R binds to I*κ*B*α*, p65, and p50 *in vitro*. The recombinant GST-tagged pS273R was incubated with the HA-tagged I*κ*B*α*, p65, or p50, followed by western blotting using the indicated antibodies. (**E**) The ASFV pS273R interacts with the endogenous I*κ*B*α*, p65, or p50. PAMs were infected with ASFV-WT. At 24 hpi, the cells were lysed for co-IP using anti-pS273R PAb, followed by western blotting analysis using the indicated antibodies. (**F**) The ASFV pS273R is colocalized with I*κ*B*α*, p65, or p50 mainly in the cytoplasm. PAMs were infected with ASFV-WT. At 24 hpi, the cells were fixed with 4% paraformaldehyde. I*κ*B*α*, p65, p50, and pS273R were immunoblotted using anti-I*κ*B*α*, -p65, -p50, and -pS273R antibodies, respectively. The nuclei were stained with DAPI and subjected to confocal microscopy analysis. The colocalization of pS273R and the molecules tested was analyzed using the Coloc2 tool of ImageJ and shown as Pearson’s correlation coefficients. Scale bar = 10 µm.

To identify the targets involved in pS273R function, various components of the canonical NF-*κ*B signaling pathway (including TRADD, MYD88, TRAF6, TAK1, TAB1, IKK*β*, and p65) were coexpressed with pS273R in HEK293T cells. The reporter assay revealed that pS273R inhibited the activation of the NF-*κ*B promoter mediated by all the tested molecules upstream of p65 (Fig. 3A), indicating that pS273R regulates the activation of the NF-*κ*B promoter specifically at the p65 level. On the other hand, considering that pS273R has an unbiased influence on the TNF-*α*- or IL-1*β*-triggered NF-*κ*B signaling pathway, we presume that pS273R probably targets the TAK1/TABs complex or its downstream proteins. The co-IP assay showed that pS273R interacted with I*κ*B*α*, p65, and p50, but not with TAK1, TAB1, or IKK*β* (Fig. 3B and C). The GST pulldown assay further confirmed that pS273R directly bound to I*κ*B*α*, p65, and p50 *in vitro* (Fig. 3D). In addition, endogenous co-IP assay indicated that ASFV pS273R was constitutively associated with I*κ*B*α*, p65, and p50 in PAMs (Fig. 3E). Consistently, confocal microscopy showed that ASFV pS273R was mainly colocalized with I*κ*B*α*, p65, and p50 in the cytoplasm (Fig. 3F). These results suggest that pS273R mainly targets the NF-*κ*B complex to regulate the activation of the NF-*κ*B signaling pathway.

### The ASFV pS273R does not affect the transcription, ubiquitination, and SUMOylation of IκBα

**Fig 4:**
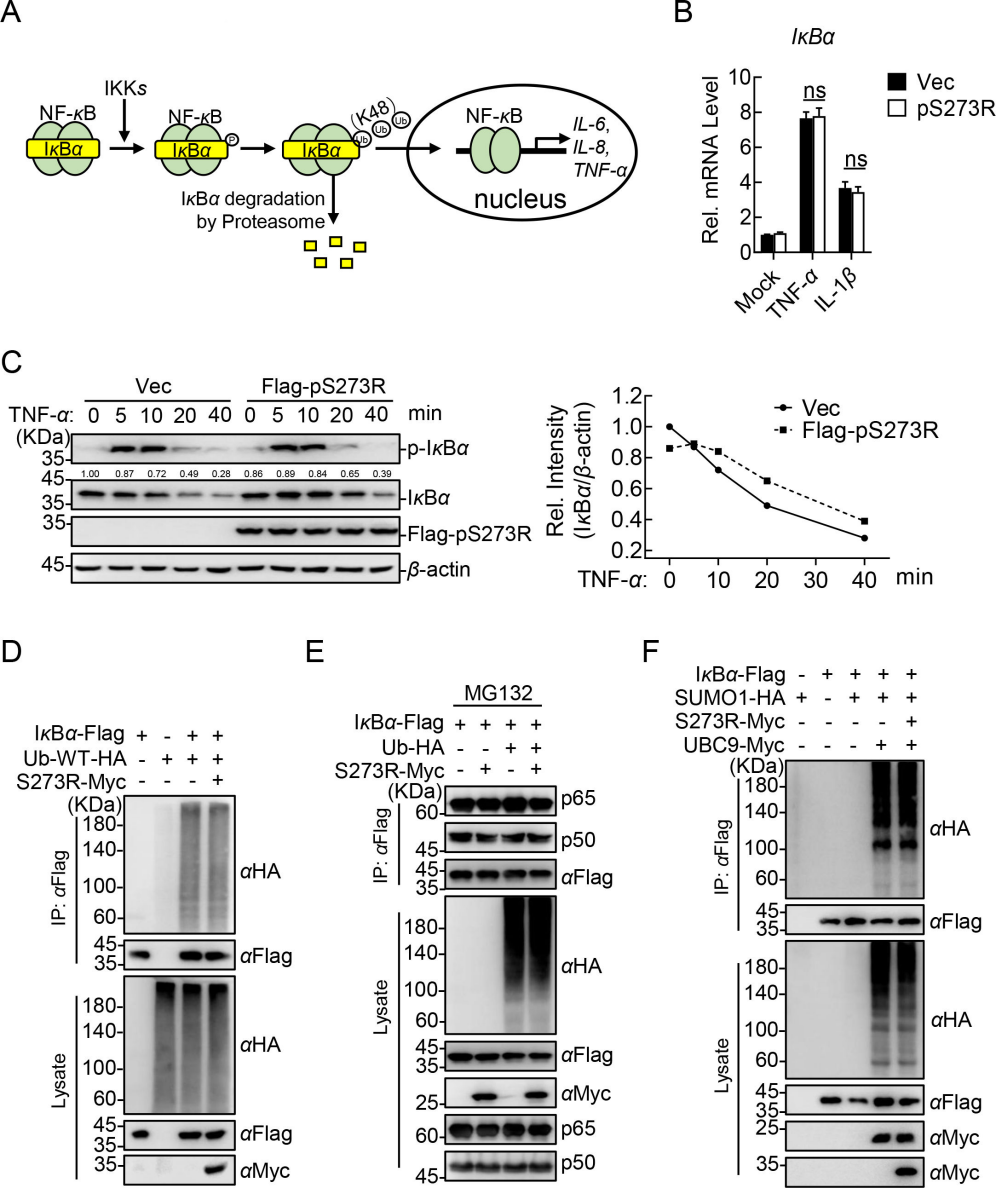
The ASFV pS273R does not affect the transcription, ubiquitination, and SUMOylation of I*κ*B*α*. (**A**) The diagram of the dynamic regulation of I*κ*B*α* by IKK*β*. (**B**) The ASFV pS273R does not impair the transcription of I*κ*B*α*. HEK293T cells were transfected with pFlag-S273R or pRK (Vec) and then treated with 20 ng/mL TNF-*α* or IL-1*β* at various times. The total RNA was extracted for RT-qPCR assay. (**C**) The ASFV pS273R significantly suppresses the degradation of I*κ*B*α* induced by TNF-*α*. HEK293T cells were transfected with pFlag-S273R or Vec. The cells were then treated with TNF-*α* at the indicated hpt, followed by western blotting analysis using the indicated antibodies. The densitometric analysis of the protein expression levels was performed using ImageJ software. Ratio: I*κ*B*α*/*β*-actin. (**D**) The ASFV pS273R does not affect the polyubiquitination of I*κ*B*α*. HEK293T cells were cotransfected with the HA-Ub-WT-, Flag-I*κ*B*α*-, and Myc-pS273R-expressing plasmids, followed by co-IP and western blotting analysis using the indicated antibodies. (**E**) The ASFV pS273R does not affect the interaction between ubiquitinated I*κ*B*α* and p65-p50. HEK293T cells were cotransfected with the plasmids expressing HA-Ub, Flag-I*κ*B*α*, and Myc-pS273R and treated with MG132*,* followed by co-IP and western blotting analysis using the indicated antibodies. (**F**) The ASFV pS273R does not affect the SUMOylation of I*κ*B*α*. HEK293T cells were cotransfected with the HA-SUMO1-, Flag-I*κ*B*α*-, Myc-UBC9-, and Myc-pS273R-expressing plasmids, followed by co-IP and western blotting using the indicated antibodies. The data shown were the means ± SDs from one representative experiment performed in triplicates (**B**). *P < 0.05; **P < 0.01; ***P < 0.001; and ns, not significant (*P* > 0.05) (unpaired Student’s *t* test).

I*κ*B*α* plays a central role in activation of the canonical NF-*κ*B signaling pathway triggered by TNF-*α*. As illustrated in Fig. 4A, once signaling proceeds downward, I*κ*B*α* is phosphorylated and activated by IKK*β*, followed by I*κ*B*α* ubiquitination and degradation by the proteasome pathway, leading to the release of the p65-p50 heterodimer (32). In this study, we found that pS273R could significantly delay the degradation of I*κ*B*α*; thus, we hypothesized that I*κ*B*α* was the main target regulated by pS273R. However, the RT-qPCR results indicated that overexpression of pS273R had no effect on the mRNA level of *IκBα*, with or without TNF-*α* treatment (Fig. 4B). Meanwhile, western blotting analysis revealed that pS273R significantly suppressed the degradation of I*κ*B*α* induced by TNF-*α* (Fig. 4C), indicating that pS273R is involved in stabilizing I*κ*B*α*.

To clarify how pS273R regulates the stability of I*κ*B*α*, the ubiquitination of I*κ*B*α* was examined first, and we found that pS273R had no effect on the polyubiquitination of I*κ*B*α* (Fig. 4D). Meanwhile, the interaction of ubiquitinated I*κ*B*α* and p65-p50 in the presence of MG132 and the effect of pS273R on the interaction were detected. The results showed that the ubiquitination of I*κ*B*α* had no effect on its interaction with p65-p50 under MG132 treatment. Additionally, pS273R also did not affect the interaction between ubiquitinated I*κ*B*α* and p65-p50. Collectively, pS273R does not exert the function via enhancing the stability of the ubiquitinated I*κ*B*α*-p65-p50 complex (Fig. 4E). It has been reported that SUMO1-modified I*κ*B*α* cannot be ubiquitinated and is resistant to the proteasome-mediated degradation (33). Considering that pS273R is a specific SUMO1 cysteine protease (25), we wondered whether pS273R functions by regulating the SUMOylation of I*κ*B*α*. Western blotting analysis *in vitro* indicated that pS273R had no effect on SUMO1 modification of I*κ*B*α* (Fig. 4F). Taken together, pS273R does not affect the transcription, ubiquitination, or SUMOylation of I*κ*B*α*.

### The ASFV pS273R disturbs the association of IκBα with the proteasome

**Fig 5:**
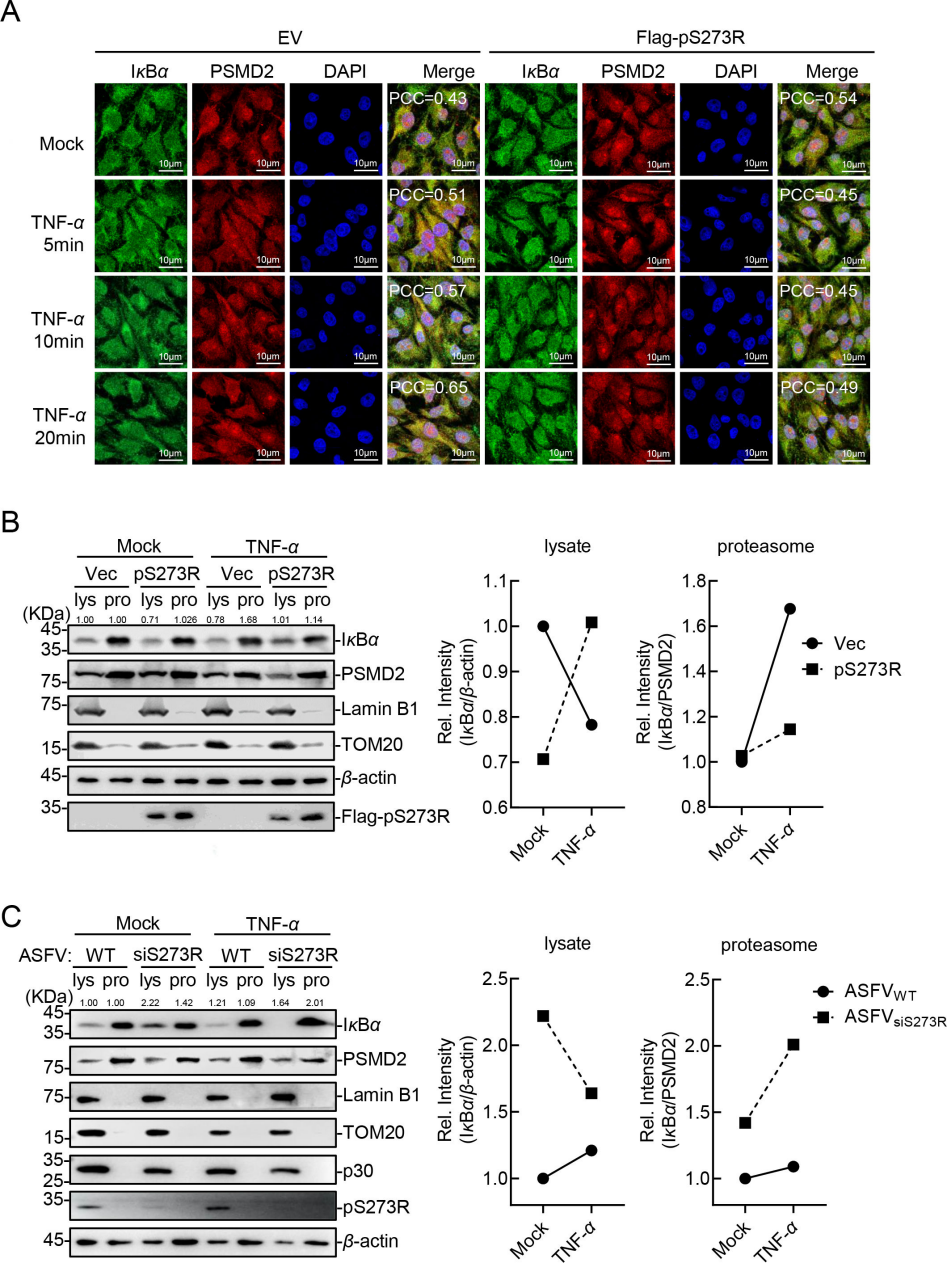
ASFV pS273R disturbs the association of I*κ*B*α* with the proteasome. (**A**) The ectopically expressed pS273R prevents the I*κ*B*α* from translocating into the proteasome from the cytoplasm. PK-15 cells stably expressing pS273R or pLOV (EV) were treated with 20 ng/mL TNF-*α* for the indicated time points and then fixed for immunoblotting and confocal microscopy using anti-I*κ*B*α* and anti-PSMD2 antibodies. The colocalization of I*κ*B*α* and PSMD2 was analyzed using the Coloc2 tool of ImageJ/FIJI and was shown as Pearson’s correlation coefficients. Scale bar = 10 µm. (**B and C**) The ASFV pS273R antagonizes the TNF-*α*-induced aggregation of I*κ*B*α* in proteasomes. HEK293T cells were transfected with pFlag-S273R or pRK (Vec), followed by treatment with TNF-*α* (20 ng/mL) (**B**). PAMs were transfected with the *S273R*-specific siRNAs and infected with ASFV and then treated with TNF-*α* (**C**). The proteasomes were isolated and subjected to western blotting analysis using the indicated antibodies. The densitometric analysis of the protein expression levels was performed using the ImageJ software. Ratio: lysate I*κ*B*α*/*β*-actin; proteasome ratio: proteasome I*κ*B*α*/PSMD2.

Since pS273R had no effect on the transcription and post-translational modifications of I*κ*B*α*, we next explored whether pS273R interferes with the last step of I*κ*B*α* degradation, namely the translocation into the proteasome. The colocalization of I*κ*B*α* with proteasome was detected by fluorescence confocal microscopy. The results showed that the I*κ*B*α* is predominantly localized in the cytoplasm, the colocalization of I*κ*B*α* with the proteasome exhibited gradual enhancement upon TNF-*α* treatment, whereas the presence of pS273R significantly impeded TNF-*α*-induced translocation of I*κ*B*α* to the proteasome (Fig. 5A). These results indicated that pS273R inhibited the degradation of I*κ*B*α* by preventing its translocation to the proteasome from the cytoplasm. Subsequently, western blotting analysis showed that pS273R remarkably inhibited the degradation of I*κ*B*α* and reduced the distribution of I*κ*B*α* in the proteasome (Fig. 5B). Furthermore, proteasome enrichment experiments after ASFV infection indicated that pS273R deficiency attenuated the ability of ASFV to antagonize the TNF-*α*-induced I*κ*B*α* aggregation in the proteasomes (Fig. 5C). In brief, pS273R inhibits the degradation of I*κ*B*α* by hindering the binding of I*κ*B*α* to the proteasome, thereby negatively regulating the activation of the canonical NF-*κ*B signaling pathway.

### The ASFV pS273R inhibits the nuclear translocation of p65

**Fig 6:**
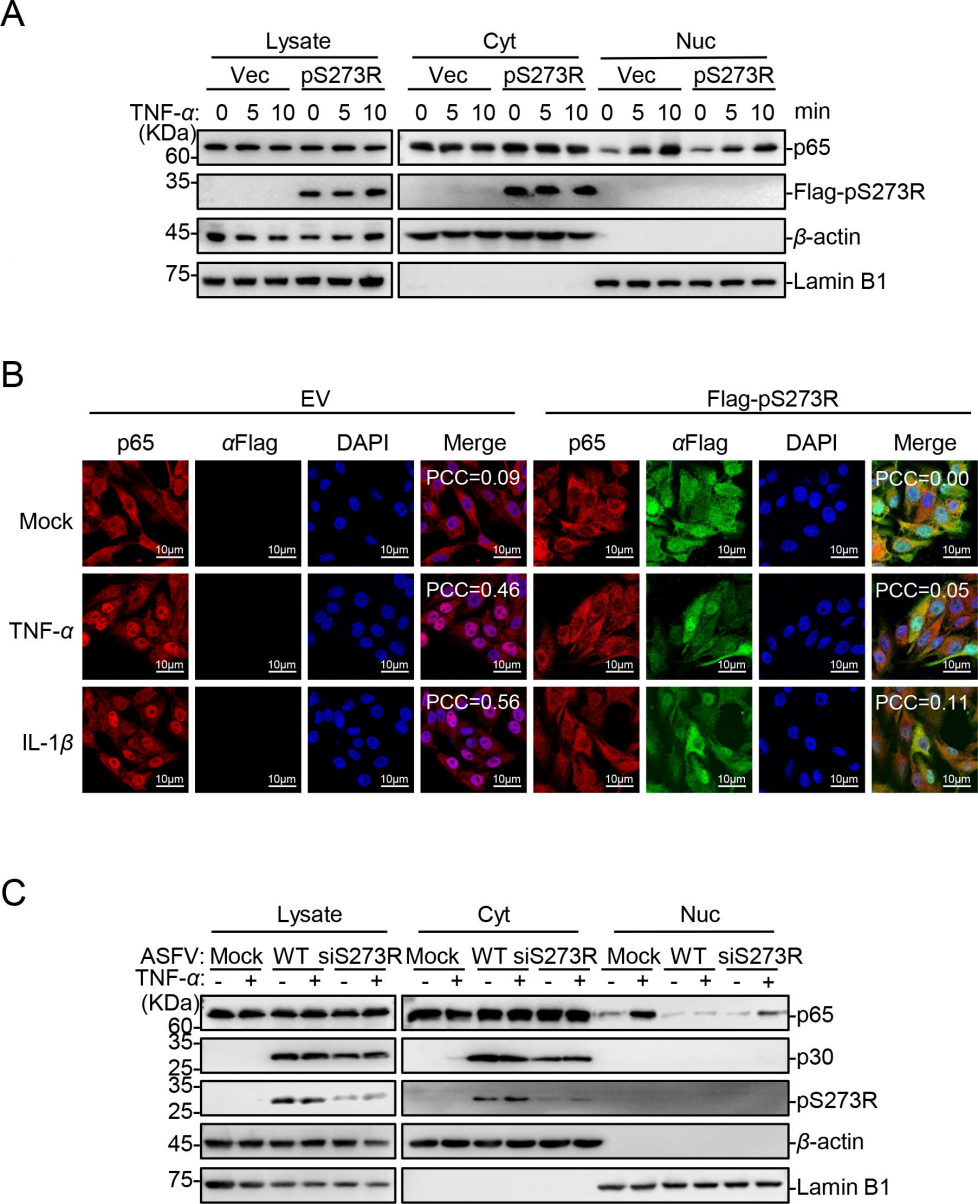
The ASFV pS273R inhibits the nuclear translocation of p65. (**A**) ASFV pS273R reduces the distribution of p65 in the nucleus. HEK293T cells were transfected with pFlag-S273R or pRK (Vec). The cells were then treated with 20 ng/mL TNF-*α* at the indicated hpt. The cells were then concentrated and lysed, followed by subcellular fractionation and western analysis blotting using the indicated antibodies. (**B**) The ASFV pS273R inhibits the nuclear translocation of p65. The stable cell lines overexpressing pS273R were treated with 20 ng/mL TNF-*α* or IL-1*β* and then fixed for immunoblotting followed by confocal microscopy analysis. The colocalization of p65 and DAPI was analyzed using the Coloc2 tool of ImageJ/FIJI and was shown as Pearson’s correlation coefficients. Scale bar = 10 µm. (**C**) Knockdown of pS273R attenuates the ability of ASFV to antagonize the TNF-*α*-induced nuclear translocation of p65. PAMs were transfected with the siRNAs for 12 h and then infected with ASFV for 24 h, followed by treatment with TNF-*α* or left untreated. The cells were then concentrated and lysed, followed by subcellular fractionation and subjected to western blotting analysis using the indicated antibodies.

As reported, the nuclear translocation of p65, which is released after I*κ*B*α* degradation, is an indicator of the canonical NF-*κ*B signaling activation (34). Therefore, we examined whether the nuclear translocation of p65 is influenced by the presence of pS273R. Cellular fractionation experiments and confocal microscopy showed that pS273R severely impeded the TNF-*α*-induced nuclear translocation of p65 (Fig. 6A and B). Moreover, knockdown of *S273R* attenuated the ability of ASFV to antagonize the TNF-*α*-induced nuclear translocation of p65 (Fig. 6C). These results indicate that the knockdown of *S273R* significantly impairs the ability of ASFV to inhibit the activation of the canonical NF-*κ*B signaling pathway.

### The amino acids (aa) 83-273 of pS273R are essential for suppressing the canonical NF-κB signaling pathway

**Fig 7:**
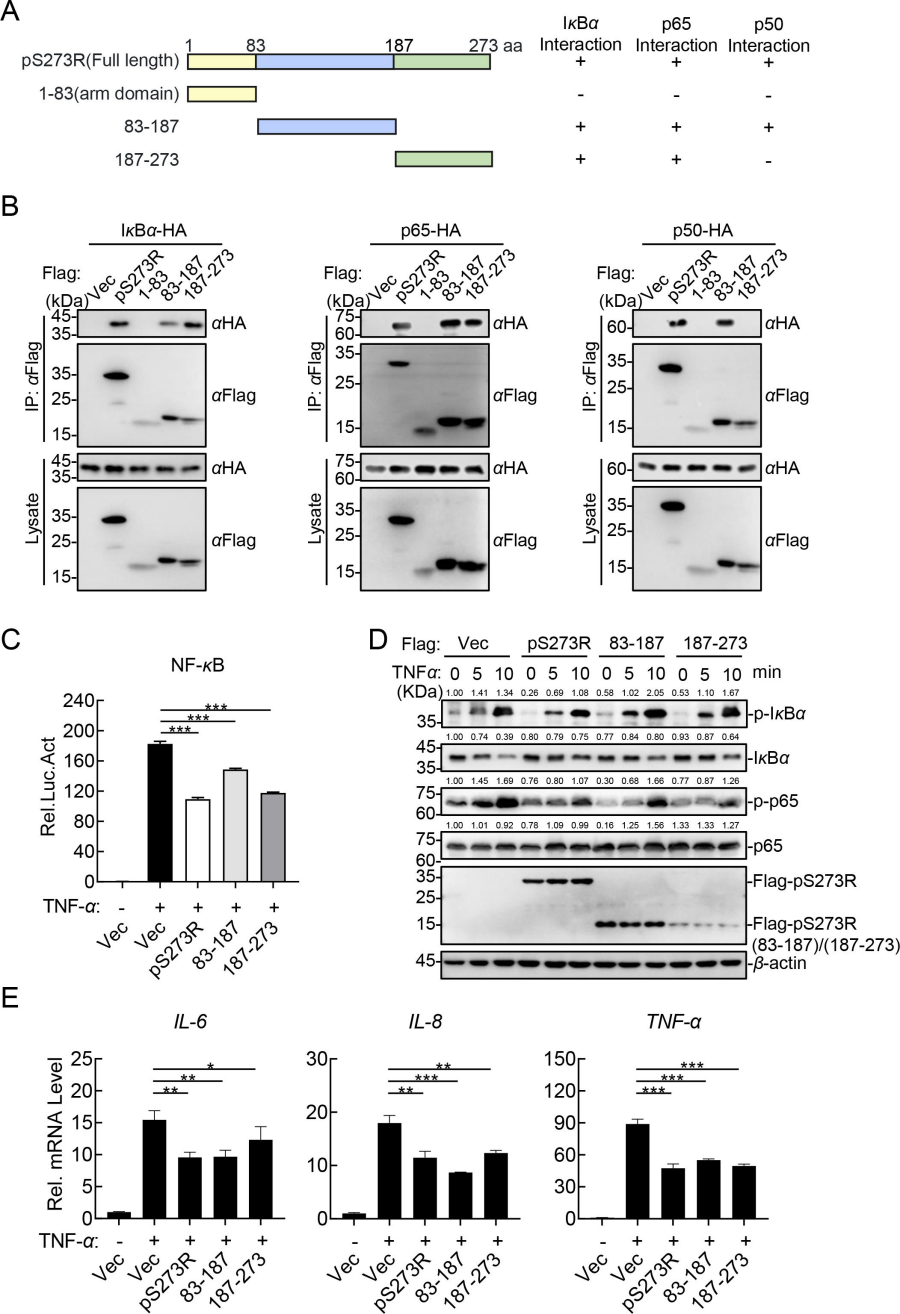
The amino acids 83–273 of the ASFV pS273R are essential for suppressing the NF-*κ*B signaling pathway. (**A**) Schematic illustration of the truncated pS273R mutants. (**B**) The different domains of pS273R interact with the NF-*κ*B complex. HEK293T cells were cotransfected with the plasmids expressing the HA-I*κ*B*α*, -p65, or -p50, and pFlag-S273R or the plasmids expressing the truncated pS273R mutants and then lysed for co-IP assay using anti-Flag MAb, followed by western blotting analysis using the indicated antibodies. (**C**) The aa 83–273 of pS273R are sufficient to inhibit the TNF-*α*-triggered activation of the NF-*κ*B promoter. HEK293T cells were cotransfected with the plasmids expressing the NF-*κ*B reporter, pRL-TK, and pFlag-S273R or the plasmids expressing the truncated versions, followed by luciferase assay. (**D**) The aa 83–273 of pS273R remarkably impaired the TNF-*α*-triggered phosphorylation of p65. HEK293T cells were transfected with pFlag-S273R or the plasmids expressing the truncated versions of pS273R and treated with 20 ng/mL TNF-*α* for the indicated time, subjected to Western blotting analysis using the indicated antibodies. The densitometric analysis of the protein expression levels was performed using ImageJ software. Ratio: target protein/*β*-actin. (**E**) The aa 83–273 of pS273R reduce the TNF-*α*-triggered transcription levels of the *IL-6*, *IL-8*, and *TNF-α* genes. HEK293T cells were transfected with pFlag-S273R or the plasmids expressing the truncated versions and treated with 20 ng/mL TNF-*α*, and then the total RNA was isolated for the RT-qPCR assay. The data shown were the means ± SDs from one representative experiment performed in triplicates (**C and E**). ***, *P* < 0.001 (unpaired Student’s *t* test).

To investigate which domain of pS273R is responsible for its association with the NF-*κ*B complex, three plasmids expressing the truncated pS273R mutants were constructed (Fig. 7A) (26). According to the domain mapping analysis of pS273R, the amino acids (aa) 83–187 were responsible for its interaction with I*κ*B*α*, p65, and p50, and the regions aa 187–27 were essential for its interaction with I*κ*B*α* and p65 (Fig. 7B). The function is determined by the protein structure; we next determined whether pS273R(aa83-187) and pS273R(aa187-273) are sufficient to inhibit the TNF-*α*-triggered NF-*κ*B signaling pathway activation. The reporter assay results showed that both pS273R(aa83-187) and pS273R(aa187-273) efficiently inhibited the TNF-*α*-triggered activation of the NF-*κ*B promoter (Fig. 7C). Western blotting analysis showed that the phosphorylation of p65 was remarkably impaired in both the pS273R(aa83-187)- and pS273R(aa187-273)-overexpressing HEK293T cells (Fig. 7D). Consistently, overexpression of pS273R(aa83-187) or pS273R(aa187-273) significantly reduced the transcription levels of the *IL-6*, *IL-8*, and *TNF-α* genes in HEK293T cells (Fig. 7E). Overall, our results suggest that the core domain (aa 83–273) of pS273R is essential for the suppression of the canonical NF-*κ*B signaling pathway.

## DISCUSSION

Vaccination is the most effective means of epidemic prevention and control. However, due to the limited understanding of the mechanism of ASFV infection and immune evasion, the development of vaccines against ASF as well as therapeutics to treat the disease are still unattainable (35). As a structural protein of ASFV, pS273R plays an indispensable role in viral replication and proliferation and is also involved in the regulation of various host physiological activities, including cell cycle, stress response, and antiviral immunity. Inflammatory responses, especially the canonical NF-*κ*B signaling pathway, play important roles in ASFV infection and pathogenesis. This study fills the gap in understanding the relationship between pS273R and inflammation regulation by identifying the ASFV pS273R as an antagonist of the canonical NF-*κ*B signaling pathway. This finding enhances our understanding of the immunosuppressive activity of pS273R and provides novel insights into ASFV biological characteristics.

ASFV pS273R not only plays a crucial role in regulating the host immune response but is also essential for viral maturation and infectivity, as it cleaves pp220 and pp62 (25), producing p5, p14, p34, p37, and p150 from pp220, and p8, p15, and p35 from pp62, respectively (36). In this study, we observed that pS273R plays an important role in the ASFV immunoevasion. Considering that pS273R functions unbiasedly in the TNF-*α*- or IL-1*β*-triggered NF-*κ*B signaling pathway, we hypothesized that pS273R functions at the level of or downstream of the TAK1-TABs complex, which is the convergence of two pathways (37). Consistently, pS273R inhibited the activation of the NF-*κ*B promoter mediated by all the tested molecules upstream of p65, indicating that pS273R functions at the NF-*κ*B complex. Meanwhile, we observed that pS273R increased the stability of I*κ*B*α* significantly among different immunoblotting analyses, indicating that pS273R may mainly target I*κ*B*α*, the negative regulatory subunit of NF-*κ*B, to participate in the regulation of the canonical NF-*κ*B signaling pathway activation.

ASFV pS273R has been reported to regulate various intracellular mechanisms through its cysteine enzyme activity. More specifically, pS273R serves as a prominent negative regulator of the cGAS-STING pathway by targeting IKK*ε* via its enzymatic activity (28). Moreover, pS273R facilitates viral replication by cleaving the nucleating protein G3BP1 to inhibit the formation of stress granules (30). Through functional verification of pS273R-3M, we have confirmed that the inhibitory effect of inflammatory response is independent of the enzymatic activity of pS273R. It has been reported that post-translational modifications, especially ubiquitination and SUMOylation, directly control the activation and degradation of I*κ*B*α*. ASFV MGF300-4L interacts with I*κ*B*α* to reduce the ubiquitination of I*κ*B*α*, thereby inhibiting the degradation of I*κ*B*α* that is dependent on ubiquitination (19). Our results demonstrated that pS273R blocked the binding of I*κ*B*α* to proteasomes. Ubiquitinated I*κ*B*α* is degraded through the proteasome pathway (38). However, the details of I*κ*B*α* recognized by the proteasome are still unclear. The intrinsic ubiquitin receptors, including proteasome regulatory particle base subunits 1, 10, and 13 (Rpn1, Rpn10, and Rpn13), determine the capability of the proteasome to recognize the ubiquitin chain and thus provide selectivity for the proteasome (39, 40). It has been shown that I*κ*B*α* can be directly degraded by the 20S proteasome, and this degradation is likely to be mediated, at least in part, by interaction with *α*2 proteasomal subunit (41). Chaperone of proteasome assembly PSMD9 interacts with I*κ*B*α via* heterogeneous nuclear ribonucleoprotein A1 (hnRNPA1), thereby enhancing the proteasomal degradation of I*κ*B*α* (42). Whether and how pS273R cooperates with ubiquitin receptors or proteasomal subunit in regulating the proteasome degradation of I*κ*B*α* requires further investigation.

**Fig 8:**
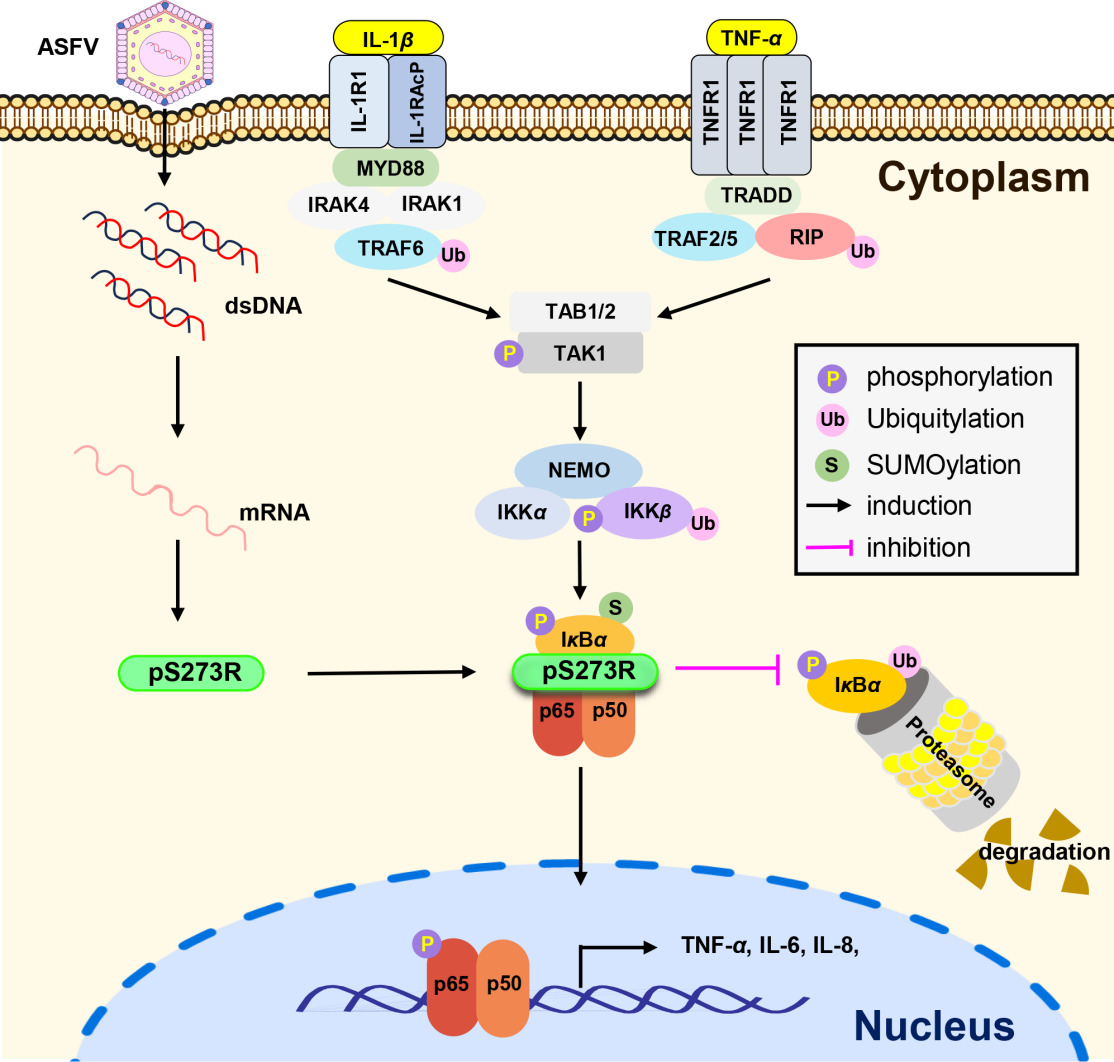
Schematic diagram of the mechanism by which the ASFV pS273R suppresses host inflammatory responses. The ASFV pS273R inhibits the TNF-*α*- or IL-1*β*-triggered inflammatory responses by targeting I*κ*B*α*. pS273R inhibits the degradation of I*κ*B*α*by disturbing the association of I*κ*B*α* with the proteasome, leading to the reduced phosphorylation and nuclear translocation of p65 and subsequently the lower expression levels of proinflammatory cytokines.

In conclusion, as illustrated in Fig. 8, we have demonstrated a protease-independent function of the ASFV pS273R in regulating the canonical NF-*κ*B signaling pathway. The ASFV pS273R inhibits the production of proinflammatory cytokines and promotes viral immunoevasion by targeting the NF-*κ*B complex and hindering the association between I*κ*B*α* and the proteasome. However, as a giant and complex DNA virus, ASFV encodes many proteins to counteract host inflammatory response, such as pMGF505-7R, pMGF300-4L, pH240R, pI10L, and pA238L. These proteins exhibit varying levels of abundance across different stages of viral replication and interact with distinct molecular targets within the NF-*κ*B pathway during viral infection. It is possible that they orchestrate the modulation of host inflammation via finely tuned cooperative mechanisms. Our findings help clarify the complicated immunoevasion mechanisms of ASFV and provide insights into the development of novel antiviral targeting pS273R.

## MATERIALS AND METHODS

### Biosafety statement and facilities

All the experiments with live ASFVs were conducted within the animal biosafety level 3 (ABSL-3) facilities at the Harbin Veterinary Research Institute (HVRI) of the Chinese Academy of Agricultural Sciences (CAAS).

### Cells and viruses

PAMs were isolated from the lung lavage fluid of 4-week-old healthy specific pathogen-free piglets and maintained in an RPMI-1640 medium containing 10% fetal bovine serum (FBS) (Gibco, Grand Island, USA), 100 U/mL penicillin, and 50 mg/mL streptomycin (Gibco, Grand Island, USA). HEK293T cells were kindly provided by Prof. Hong-Bing Shu and PK-15 cells were obtained from the American Type Culture Collection. HEK293T and PK-15 cells were cultured in Dulbecco’s modified Eagle’s medium (DMEM) supplemented with 10% FBS. The ASFV HLJ/2018 strain (ASFV-WT) (GenBank accession no. MK333180.1) was isolated from field samples in China as described previously (43).

Stable cell lines overexpressing pS273R or its mutant pS273R-3M were constructed using the lentivirus-mediated gene-editing technology (21). Briefly, HEK293T cells were cotransfected with the packaging plasmids psPAX2 and pMD2.0G, the donor plasmid pLOV-S273R, pLOV-pS273R-3M, or the pLOV vector. At 48 h of post-transfection (hpt), the recombinant virus-containing culture supernatants were harvested and transduced into PK-15 cells in the presence of 8 µg/mL polybrene. A stable cell line was screened out after cultured three passages of the transduced cells in the medium supplemented with 3 µg/mL puromycin. Finally, western blotting and confocal microscopy were performed to identify the expression of pS273R.

### Reagents and antibodies

The recombinant human IL-1*β* (catalog no. 200–01B) and TNF-*α* (catalog no. 300–01A) were purchased from PeproTech (Cranbury, NJ, USA). The dual-luciferase reporter assay system (catalog no. E1960) was purchased from Promega (Madison, WI, USA). The Minute cytosolic proteasome enrichment kit (catalog no. PT-040) was purchased from Invitrogen (Beijing, China). IL-1*β* ELISA kit (catalog no. ELP-IL1b) was purchased from Raybiotech (California, USA). The TRIzol reagent (catalog no. 9108), HiScript III 1st strand cDNA synthesis kit (catalog no. R312-01), and HiScript II Q RT SuperMix (catalog no. R223-01) were purchased from Vazyme (Nanjing, China). Polyethylenimine (catalog no. 24765) was purchased from PolySciences (Warrington, USA). Anti-Flag M2 Magnetic Beads (catalog no. M8823) were purchased from Sigma. The ChromoTek GST-Trap agarose beads (catalog no. sta-20) were purchased from Proteintech (Chicago, IL, USA).

The following antibodies were purchased from Proteintech: rabbit monoclonal antibody (MAb) against HA (catalog no. 66006–2-Ig) and rabbit MAb against GST (catalog no. 10000–0-AP); rabbit polyclonal antibody (PAb) against the lamin B1 (catalog no. 12987–1-AP), rabbit PAb against PSMD2 (catalog no. 14748–1-AP), and rabbit PAb against TOMM20 (catalog no. 11802–1-AP); and mouse MAb against DYKDDDDK (catalog no. 66008–4-Ig), mouse MAb against Myc (catalog no. 60003–2-Ig), and mouse MAb against *β*-actin (catalog no. CL594-66009). Rabbit MAb against the NF-*κ*B p65 (catalog no. 8242S), rabbit MAb against phospho-NF-*κ*B p65Ser536 (catalog no. 3033S), mouse MAb against I*κ*B*α* (catalog no. 4814S), and rabbit MAb against phospho-I*κ*B*α*Ser32 (catalog no. 2859S) were purchased from Cell Signaling (Danvers, MA, USA). Goat anti-mouse IgG DyLight 488 (catalog no. A23210) and goat anti-rabbit IgG DyLight 594 (catalog no. A23420) were purchased from Abbkine (Wuhan, China). 4’,6-Diamidino2-phenylindole (DAPI) (catalog no. C0065) was purchased from Solarbio (Beijing, China). Mouse anti-ASFV-p30 MAb were produced and stored in our laboratory (19). Rabbit against ASFV-pS273R PAb was kindly provided by Professor Yan-Yi Wang from the Wuhan Institute of Virology, Chinese Academy of Sciences. Rabbit against ASFV-pS273R MAb were kindly provided by Professor Jianzhong Zhu from College Veterinary Medicine, Yangzhou University. Mouse against ASFV-pS273R MAb were kindly provided by Professor Changjiang Weng from Harbin Veterinary Research Institute of the Chinese Academy of Agricultural Sciences.

### Plasmid construction

The recombinant plasmids pRK-Flag-pS273R, pRK-TRADD-HA, pRK-MYD88-HA, pRK-TRAF6-HA, pRK-TAK1-HA, pRK-TAB1-HA, pRK-IKK*β*-HA, pRK-I*κ*B*α*-HA, pRK-p65-HA, and pRK-p50-HA were described previously (21). The plasmids pNF-*κ*B-Fluc, pRL-TK, psPAX2, and pMD2.0G, and the pLOV vector were kindly provided by Prof. Hong-Bing Shu. The plasmid expressing SUMO1 was a gift from Dr. Yina Zhang at Henan Agriculture University. The plasmids pRK-Flag-pS273R-3M (H168R/N187A/C232S) and pLOV-pS273R-3M expressing the pS273R mutants were constructed using standard molecular biology techniques.

### Dual-luciferase reporter assay

HEK293T cells were transfected using polyethylenimine. HEK293T cells grown in 48-well plates were cotransfected with the reporter plasmids pNF-*κ*B-Fluc (0.1 µg/well) and pRL-TK (0.01 µg/well), together with pFlag-S273R or pRK (10 ng-40ng/well). At 20 hpt, the cells were treated with TNF-*α* or IL-1*β* for 10 h and harvested using a passive lysis buffer. Luciferase assay was performed using a dual-specific luciferase assay kit (Promega, Wisconsin, USA). The *firefly* luciferase activities were normalized on the basis of the *renilla* luciferase activities. The data were shown as the means from representative experiments in triplicates.

### Reverse transcription-quantitative PCR (RT-qPCR)

Total RNA was extracted from the transfected cells in 12-well plates using the TRIzol reagent and reverse-transcribed into cDNA using the HiScript III 1st strand cDNA synthesis kit according to the manufacturer’s protocols. RT-qPCR was performed using HiScript II Q RT SuperMix according to the manufacturer’s protocol. The data were shown as the relative abundance of the indicated mRNA normalized to that of *GAPDH*. The primers used for RT-qPCR are shown in Table 1.

**TABLE 1:** Primers used for RT-qPCR

Name	Sequence (5′−3′)
hum-*GAPDH*-F	GACAAGCTTCCCGTTCTCAG
hum-*GAPDH*-R	GAGTCAACGGATTTGGTCGT
hum-*IL-6*-F	TTCTCCACAAGCGCCTTCGGTC
hum-*IL-6*-R	TCTGTGTGGGGCGGCTACATCT
hum-*IL-8*-F	GAGAGTGATTGAGAGTGGACCAC
hum-*IL-8*-R	CACAACCCTCTGCACCCAGTTT
hum-*TNF-α*-F	GCCGCATCGCCGTCTCCTAC
hum-*TNF-α*-R	CCTCAGCCCCCTCTGGGGTC
sus-*GAPDH*-F	ACATGGCCTCCAAGGAGTAAGA
sus-*GAPDH*-R	GATCGAGTTGGGGCTGTGACT
sus-*IL-6*-F	GACCCTGAGGCAAAAGGGAA
sus-*IL-6*-R	TCGTTCTGTGACTGCAGCTT
sus-*IL-8*-F	TGGCAGTTTTCCTGCTTTCT
sus-*IL-8*-R	CAGTGGGGTCCACTCTCAAT
sus-*TNF-α*-F	GCCCAAGGACTCAGATCATC
sus-*TNF-α*-R	GGCATTGGCATACCCACTCT
ASFV-*S273R*-F	GGACTTTTCAACGGGCACTG
ASFV-*S273R*-R	ATAGCTGCTGTTTGACCCGT

### Confocal imaging

Confocal imaging was performed as described previously (44). Briefly, at 24 hpt, the HEK293T cells were fixed with 4% paraformaldehyde for 30 min and permeabilized for 20 min with 0.1% Triton X-100. The cells were blocked with 1% bovine serum albumin for 30 min and then incubated with the indicated antibodies and corresponding dye-conjugated secondary antibodies for 1 h at 37°C. Finally, the cells were stained with DAPI for 10 min. The cells were imaged under a Zeiss confocal microscope under a 63 × oil-immersion lens.

### Coimmunoprecipitation (Co-IP) assay

Co-IP and western blotting analyses were performed as described previously (45). The cells were lysed with the M2 lysis buffer (20 mM Tris-HCl [pH 7.5], 0.5% NP-40, 10 mM NaCl, 3 mM EDTA, and 3 mM EGTA) containing protease inhibitors and sonicated for 2 min. The lysates were centrifuged at 13,000 × *g* for 10 min at 4°C. The supernatants were immunoprecipitated with the indicated antibodies or anti-Flag M2 magnetic beads for 4 h. The beads were then washed three times with the high-salinity M2 lysis buffer (0.5 M NaCl). The bound proteins were separated using sodium dodecyl sulfate-polyacrylamide gel electrophoresis (SDS-PAGE), followed by western blotting analysis with the indicated antibodies.

### GST pulldown assay

GST pulldown assay was performed as described previously (21). To prepare *Escherichia coli* for pS273R expression, the recombinant plasmid pGEX-6p-1-pS273R was transformed into *E. coli* BL21 cells. Then, the *E. coli* BL21 cells with optical density (OD) 600 nm values between 0.4 and 0.6 were treated with 1 mM isopropyl *β*-D-thiogalactoside (IPTG) for 12 h at 16°C. After centrifugation, the enriched bacterial cultures were resuspended and lysed using a high-pressure homogeneous sterilizer. The supernatants containing the recombinant GST or GST-pS273R were purified using the ChromoTek GST-Trap agarose beads. GST and GST-pS273R were incubated with the lysates of the HEK293T cells containing the ectopically expressed I*κ*B*α*, p65, or p50 at 4°C for 12 h. Subsequently, western blotting analysis was performed using the indicated antibodies.

### Subcellular fractionation assay

To separate the nuclear fraction (Nuc) and cytoplasmic fraction (Cyt), the cells were washed three times with PBS and once in hypotonic buffer (10 mM Tris-HCl pH 7.4, 10 mM KCl, 1.5 mM MgCl_2_) supplemented with protease inhibitor, resuspended in hypotonic buffer, and lysed by leaching homogenization. The lysates were centrifuged at 4°C for 10 min at 500 × *g* to separate the sediment of Nuc, and the supernatants were collected as Cyt. The Nuc was resuspended in RIPA buffer containing an equal volume of Cyt, and western blotting analysis was performed using the indicated antibodies.

### RNA interference (RNAi)

Small interfering RNAs (siRNAs) targeting the ASFV *S273R* gene and non-targeting siRNA (siNC) were chemically synthesized (GenePharma, Shanghai, China). Briefly, PAMs were transfected with the corresponding siRNAs using the Lipofectamine RNAiMAX transfection reagent (Invitrogen, Carlsbad, CA, USA) according to the manufacturer’s instructions. At 12 hpt, the cells were infected with ASFV at a multiplicity of infection of 1 for 24 h. The cells were collected, and the knockdown efficiency was analyzed by RT-PCR.

The following sequences were targeted for the *S273R* mRNA: siRNA#1: 5′-GCGUUUUAAACACGGACUUTT-3′; siRNA#2: 5′-GCAAAGUGGUCAAGCGUCC UTT-3′; siRNA#3: 5′-GCGAUUCGGAGUCCUGCGUTT-3′.

### Proteasome isolation

HEK293T cells grown in 6-well plates were transfected with pFlag-pS273R or pRK. At 24 hpt, the cells were treated with TNF-*α* (20 ng/mL) for 10 min. The cell cultures were enriched, and the Minute cytosolic proteasome enrichment kit was used for proteasome enrichment according to the manufacturer’s protocols. Finally, the pellets were dissolved in the protein-loading buffer and subjected to western blotting analysis.

### Statistical analysis

GraphPad Prism and SPSS Statistics were used for statistical analysis. The quantitative data in histograms were shown as the means ± standard deviations. The data were analyzed using the unpaired Student’s *t* test. The number of asterisks represents the degree of significance of the *P* values. Statistical significance was set at *P* values < 0.05. *P* values were indicated by asterisks in the Figures as follows: *P < 0.05, **P < 0.01, and ***P < 0.001.

## Data Availability

All data have been included in the article. All reported data and any additional information required to reanalyze the data are available from the lead contact upon reasonable request.
